# Advancements in the understanding of mechanisms of the IL-6 family in relation to metabolic-associated fatty liver disease

**DOI:** 10.3389/fendo.2025.1642436

**Published:** 2025-09-03

**Authors:** Jiale Liu, Tao Wang, Fuyuan Yang, Ciren Pubu

**Affiliations:** ^1^ Department of Anaesthesiology, The First Affiliated Hospital of Yangtze University, Jingzhou, Hubei, China; ^2^ School of Basic Medicine, Yangtze University Health Science Center, Jingzhou, Hubei, China; ^3^ Shannan Maternal and Child Health Hospital, Shannan, China

**Keywords:** IL-6 family, metabolic-associated fatty liver disease, lipid metabolism, insulin resistance, inflammation

## Abstract

As lifestyle patterns change, the rates of type 2 diabetes and obesity are increasing together, leading to an increase in metabolic-associated fatty liver disease (MAFLD), now recognized as the most frequently occurring liver disease globally. MAFLD presents a significant threat to public health and imposes a substantial socioeconomic burden. This condition encompasses a spectrum of hepatic manifestations, beginning with excessive fat accumulation and hepatic steatosis, and possibly progressing to non-alcoholic steatohepatitis (NASH), liver fibrosis, cirrhosis, and liver cancer. The pathogenesis of MAFLD is intricately linked to lipid accumulation, oxidative stress, and lipotoxicity. Notably, the interleukin-6 (IL-6) cytokine family plays a complex role in the onset and development of MAFLD, primarily through the modulation of lipid metabolism, insulin resistance, inflammatory responses, and liver fibrosis. This review examines the impact of the IL-6 family on the progression of MAFLD. It explores targeting the IL-6 family as a potential future therapy for MAFLD.

## 1 Introduction

The global epidemic and its concomitant metabolic syndrome have intensified the complexity of metabolic dysregulation, thereby contributing to the rising prevalence of diabetes and obesity-related metabolic disorders. MAFLD has emerged as the most widespread persistent liver, condition, impacting nearly a quarter of the people worldwide. As a result, it has emerged as a critical public health problem across the globe (1). MAFLD is a chronic liver condition intricately linked to obesity, diabetes mellitus, and metabolic syndrome. It is characterized as a chronic liver disease that, alongside ethanol and other definitive liver-damaging factors, appears as a clinicopathological syndrome chiefly identified by the excessive storage of fats in hepatocytes. This condition represents an acquired metabolic stress-induced liver injury, closely associated with insulin resistance and genetic predisposition (2). MAFLD encompasses a range of disorders, including Non-Alcoholic Fatty Liver (NAFL) and NASH, with the latter frequently linked to cellular fibrosis and potential progression to cirrhosis (3). In economically advanced areas such as Europe, the United States, and rich regions of China, non-alcoholic steatohepatitis has emerged as a primary factor in chronic hepatic disorder. Among the general adult population, MAFLD is present in 10% to 30% of people, with 10% to 20% of these cases being NASH, which carries a cirrhosis incidence rate of up to 25% within a decade (4).

Over the past few years, the function of IL-6 in MAFLD has emerged as a focal point in the fields of immunology and liver disease research. The IL-6 family comprises a series of cytokines predominantly produced by leukocytes, with IL-6 as the principal member. This family also includes interleukin-11 (IL-11), interleukin-27 (IL-27), interleukin-31 (IL-31), oncostatin M (OSM), leukemia inhibitory factor (LIF), ciliary neurotrophic factor (CNTF), cardiotrophin 1 (CTF1), and cardiotrophin-like cytokine factor 1 (CLCF1), among others. These cytokines are essential in regulating immune responses, inflammation, and metabolic processes (5). Research has indicated that the IL-6 family exerts multifaceted influences on the pathogenesis and progression of MAFLD, primarily by modulating hepatic immune responses, metabolic pathways, and fibrosis (6). This paper reviews the role of IL-6 family members in MAFLD development, offering insights for clinical management and early intervention.

## 2 IL-6 family members overview

The IL-6 family comprises a diverse group of cytokines that, while sharing some overlapping biological activities, also demonstrate distinct functional differences. Collectively, these cytokines play a significant role in the pathological processes associated with inflammatory responses, the regulation of lipid metabolism, fibrosis, and tumorigenesis (7). Cytokines in this family exhibit similar helical structures (8), encompassing 10 ligands and 9 receptors (9). The cytokines of the IL-6 family, along with their specific receptors, share a conserved signal transduction core protein known as gp130, which is encoded by the IL6ST, gene and forms a polymeric complex through its aggregation (10). The receptor complexes for each cytokine incorporate either one or two gp130 signaling receptor subunits (7). About target cells, IL-6 engages with the signaling subunit gp130, either in its membrane-bound state or in association with soluble IL-6 receptors, to initiate intracellular signaling pathways (11). Studies suggest that the activation of IL-6 signaling via gp130 is predominantly mediated by the Janus kinase-signal transducer and activator of transcription 3 (JAK/STAT3) signaling pathway. In this pathway, JAK1 facilitates the phosphorylation and dimerization of STAT3, which subsequently translocates to the nucleus to execute transcriptional functions (12).

### 2.1 The IL-6 family and inflammation

IL-6, a critical inducer of B-cell immunoglobulin, is predominantly expressed in lymphocytes, hepatocytes, epithelial cells, and macrophages. It is swiftly upregulated in response to infection or tissue injury, thereby facilitating the body’s defense mechanisms through the mediation of acute-phase responses, immune responses, and hematopoietic regulation (13). Furthermore, IL-6 is involved in systemic metabolic regulation, and can initiate inflammatory cascades. Empirical research has substantiated its pivotal role in various immune regulatory pathways (14). Additionally, IL-6 is typically recognized by pattern recognition receptors on immune cells at sites of infection, mediating the activation of the nuclear factor kappa-light-chain-enhancer of activated B cells(NF-κB) signaling pathway, which in turn enhances the expression of inflammatory factors, including IL-6 itself. Tumor necrosis factor-alpha (TNF-α) and interleukin-1beta (IL-1β) enhance IL-6 production by activating transcription factors, leading to a self-sustaining positive feedback loop in the inflammatory process (13).

IL-11, a cytokine secreted by fibroblasts, supports the maintenance of IL-6-dependent hematopoietic cell lines primarily through its interaction with the gp130 and IL-11 receptor complex. Additionally, IL-11 plays a crucial role in modulating inflammatory responses by exerting significant anti-inflammatory effects (15).

IL-27 is predominantly secreted by antigen-presenting cells following stimulation of pattern recognition receptors, facilitating the proliferation of naive CD4^+^T cells and the differentiation (16). into the Th1 subtype. Research suggests that IL-27 plays a crucial role in regulating the balance between pro-inflammatory and anti-inflammatory, responses, modulating immune responses, and influencing the tumor microenvironment (17).

OSM, as a multifunctional cytokine, is synthesized by a diverse array of cell types, like activated monocytes/macrophages, T cells, dendritic cells, and neutrophils (18). Primarily serving as a growth regulator, OSM demonstrates a wide range of biological functions, particularly in the contexts of inflammation, liver fibrosis, and oncological conditions (19, 20). In the realm of inflammatory diseases, OSM is capable of activating the NF-κB and JAK/STAT3 signaling pathways, which in turn stimulate cell proliferation and the secretion of inflammatory mediators, thereby facilitating the advancement of chronic inflammation (21).

IL-31, a newly identified pro-inflammatory cytokine, is predominantly expressed in activated helper T cell populations, with a particular emphasis on the Th2 cell subset (22). Upon interacting with its specific receptor, IL-31 activates the JAK/STAT, Phosphoinositide 3-kinase/Ak strain Transforming (PI3K/AKT), and Mitogen-Activated Protein Kinase(MAPK) signaling pathways, thereby influencing a variety of immune and non-immune cells to contribute to immune responses, inflammatory diseases, and allergic reactions (23). Research shows that IL-31 can trigger the overexpression of pro-inflammatory cytokines like IL-6 and other chemokines by activating extracellular signal-regulated kinases, thereby worsening inflammation (24).

### 2.2 IL-6 family and lipid metabolism

Beyond its function in modulating the equilibrium between pro-inflammatory and anti-inflammatory responses, IL-27 also plays a crucial role in enhancing thermogenesis, preventing diet-induced obesity, and ameliorating insulin resistance. This is accomplished through its direct interaction with adipocytes, where it activates the p38 Mitogen-activated protein kinase- Peroxisome proliferator-activated receptor γ coactivator-1α (p38 MAPK-PGC-1α) signaling pathway and induces the expression of uncoupling protein 1 (UCP1), thereby facilitating thermogenesis (25).

LIF is recognized as the most versatile member of the IL-6 cytokine family (26), demonstrating biological activity across a wide range of tissues (27). Prior research has substantiated its roles, notably as a human hepatocyte growth factor and as an inhibitor of differentiation in mouse embryonic stem cells. Further investigations have elucidated its capacity to inhibit lipoprotein lipase derived from melanoma, thereby modulating systemic lipid metabolism through the suppression of lipoprotein lipase activity (28).

CNTF is among the most extensively investigated neuroprotective nutritional factors, originally identified for its role in enhancing the survival and growth of ciliary ganglion cells. It is expressed in retinal cells, neurons, and adipocytes (29). Under normal conditions, CNTF helps regulate lipid metabolism by reducing food intake and increasing energy expenditure through central nervous system metabolic pathways (30, 31).

CLCF1 not only exhibits the typical biological characteristics of the IL-6 family but also significantly stimulates the proliferation of B cells (32), primarily expressed in lymphoid tissues and involved in immune regulation (33). Recent studies have identified its expression in adipocytes and hepatocytes, where CLCF1 engages in an autocrine signaling pathway in brown adipose tissue. binds to the gp130/CNTFR/LIFR receptor complex to activate downstream JAK/STAT3 signal transduction, thereby inhibiting the expression of PGC-1α and PGC-1β, ultimately leading to a reduction in adipocyte thermogenesis (30). Under conditions of metabolic stress, the expression of this factor is markedly upregulated in both adipose tissue and the liver. Research indicates that while CLCF1 inhibits thermogenesis in adipocytes, it promotes liver metabolism, suggesting that CLCF1 plays a dual regulatory role in maintaining tissue-specific homeostasis (30, 34).

### 2.3 IL-6 family and fibrosis

Research shows that IL-11, part of the IL-6 cytokine family, has anti-inflammatory effects but also plays a key role in autoimmune disease progression, bone metabolism regulation, fibrosis, and aging (35, 36). IL-11 influences key fibrotic-related genes downstream of fibrotic mediators like TGFβ1, thereby promoting the fibrotic pathological process in tissues (37).

In the context of liver diseases, OSM enhances the expression of tissue inhibitor of metalloproteinases 1 (TIMP1), suppresses fibrinolytic activity in hepatic stellate cells, and facilitates the proliferation of myofibroblasts, thereby contributing to the pathogenesis of liver fibrosis (38).

CTF1 is multifunctional, initially noted for cardioprotection, and widely expressed in the liver, kidneys, lungs, and skeletal muscle (39, 40). Beyond its role in myocardial protection (41), recent investigations have revealed that CTF1 functions as an autophagy activator in fibroblasts and tumor-associated fibroblasts. This is accomplished through the induction of STAT3 phosphorylation and nuclear translocation, which activates the transcription of autophagy-related proteins. Furthermore, CTF1synergizes with the activation of the Adenosine Monophosphate-activated Protein Kinase-Unc-51 Like Kinase 1(AMPK/ULK1) signaling axis, leading to fibroblast activation and the progression of fibrotic pathology (42).

### 2.4 IL-6 family and other diseases

The expression levels of IL-11 exhibit an increasing trend with advancing age.IL-11 plays a regulatory role in age-associated pathological processes at both the cellular and tissue levels through the sequential activation of the Extracellular signal-Regulated kinase-AMP-Activated Protein Kinase-mechanistic Target of Rapamycin Complex1 (ERK-AMPK-mTORC1) and JAK/STAT3 signaling pathways, ultimately contributing to the induction of aging (43, 44).

Within the realm of tumor pathology, OSM has been recognized as a pro-tumorigenic factor. Research shows that OSM promotes tumor invasion and metastasis by increasing matrix metalloproteinase (MMP) expression and activating the JAK/STAT3 pathway (45).

In hepatocellular carcinoma (HCC), CLCF1 enhances CXCL6 and TGF-β expression through the Akt/ERK1/2-STAT3 pathway, promoting stem cell traits and self-renewal in cancer cells (46) (**Table 1**).

**Table 1:** Overview of IL-6 family members.

Pathophysiological process	Cytokine	Functional molecules/signal pathways	Biological Function	References
Inflammatory Response	IL-6	NF-κB	Promotes the release of pro-inflammatory cytokines and acute phase response	(13, 14)
	IL-11	–	Inhibits the production of inflammatory mediators	(15)
	IL-27	–	Bidirectional regulation: pro-inflammatory/anti-inflammatory	(16, 17)
	OSM	JAK/STAT3,NF-κB	Pro-inflammatory response	(18–21)
	IL-31	JAK/STAT,PI3K/AKT,MAPK	Pro-inflammatory response	(22–24)
Lipid Metabolism	IL-27	p38 MAPK-PGC-1α	Promotes thermogenesis in fat cells	(25)
	LIF	–	Inhibits lipid synthesis	(26–28)
	CNTF	–	Increases energy expenditure	(29–31)
	CLCF1	JAK/STAT3	Inhibits thermogenesis in fat cells	(30, 32–34)
Fibrosis	IL-11	TGFβ1	Activate the conversion of fibroblasts to myofibroblasts	(35–37)
	OSM	TIMP1	Promote excessive deposition of extracellular matrix	(38)
	CTF1	STAT3,AMPK,ULK1	Exacerbate the fibrosis process by inhibiting autophagy	(39–42)
Cellular Senescence	IL-11	ERK-AMPK-mTORC1,JAK/STAT3	Induction of cell cycle arrest and senescence-associated secretory phenotype	(43, 44)
Tumor Progression	OSM	JAK/STAT3	Promotes tumor cell invasion and metastasis	(45)
	CLCF1	Akt/ERK1/2-STAT3	Enhances self-renewal of hepatocytes in liver cancer	(46)

## 3 Pathologic alterations and physiological mechanisms associated with MAFLD

### 3.1 Pathological alterations and diagnostic criteria of MAFLD

MAFLD is defined by an abnormal buildup of fat in the liver, without other causes like heavy alcohol use. Histologically, MAFLD is classified into NAFL and NASH, with the latter representing the primary progression of MAFLD (47). The difference between NAFL and NASH is based on the pathological features observed in liver histology. NAFL is predominantly marked by steatosis, accompanied by mild lobular inflammation and hepatocellular ballooning, with or without perisinusoidal fibrosis (48). In contrast, NASH is characterized by a disease process involving inflammation and hepatocellular injury, typically associated with hepatocellular fibrosis, which can develop into cirrhosis and eventually lead to hepatocellular carcinoma (49).

Biomarkers for MAFLD are integral to three primary domains: (1) quantification of hepatic fat content for diagnostic purposes; (2) evaluation of disease severity, specifically the extent of inflammation and fibrosis; and (3) monitoring temporal changes in hepatic stiffness (50). Presently, the predominant methodologies employed for MAFLD diagnosis encompass serological assays and imaging modalities. Serological assessments usually measure liver function indicators, such as alanine aminotransferase (ALT) and aspartate aminotransferase (AST) to evaluate MAFLD progression. Elevated ALT and AST levels are not definitive indicators of MAFLD, as they can remain normal in some patients, particularly early in the disease or before significant progression (51–53). As MAFLD becomes more common and diagnostic methods improve, imaging techniques like ultrasound, computed tomography (CT), and magnetic resonance imaging (MRI) can now noninvasively detect steatosis. However, a liver biopsy is still needed for a definitive NASH diagnosis, as it assesses liver tissue for fat accumulation and fibrosis progression (54).

### 3.2 Pathophysiologic mechanisms of MAFLD

The exact cause of MAFLD remains unclear, but it may be linked to insulin resistance(IR), oxidative stress, adipokines, and gut microbiota imbalance (3).

The liver is the body’s primary metabolic hub, fat digestion and absorption, coagulation factor synthesis, bile production, and hormone regulation (55). The pathological progression of MAFLD is characterized by a “three-strike” process: steatosis, lipotoxicity, and inflammation (56). Research indicates that individuals with MAFLD often adhere to unhealthy dietary patterns, marked by a high consumption of diets rich in fats or sugars. This dietary behavior impacts the synthesis and uptake of hepatic free fatty acids (FFAs), resulting in an excessive accumulation of FFAs (57). Furthermore, insulin resistance (IR) in adipose tissue causes peripheral lipolysis and substantial mobilization of FFA from adipocytes overloaded with fat to the liver, culminating in hepatic FFA accumulation (3). The accumulation of lipids within hepatocytes represents the “first strike” in the pathogenesis of MAFLD. In individuals with severe MAFLD, hepatic function significantly declines as the extent of fatty liver severity rises, resulting in hepatic IR and elevating the risk of concomitant type 2 diabetes mellitus (58). Prolonged fat accumulation stimulates the enhanced production of pro-inflammatory cytokines, for example, TNF-α, IL-6, and IL-1β, via activation of the NF-κB signaling pathway. These inflammatory cytokines facilitate the recruitment and activation of Kupffer cells, thereby mediating inflammation in NASH (59). In NASH, FFAs serve as critical mediators of lipotoxicity, facilitating the transition of the liver from basic fat accumulation to steatoinflammation (60). The inflammatory response and hepatocyte ballooning associated with steatosis contribute to hepatocyte apoptosis. Concurrently, the activation of hepatic stellate cells (HSCs), which originate from the extracellular matrix, results in the secretion of collagen fibers, thereby initiating fibrosis and hepatic parenchymal dysfunction. This process subsequently advances to hepatic fibrosis (61). It is well-established that the liver possesses a robust capacity for self-repair. However, in instances where pathogenic factors are not entirely eradicated, this self-repair capability may exacerbate hepatic fibrosis, significantly compromising the liver’s original architecture. This deterioration can lead to the progression of cirrhosis, particularly as the severity of hepatic fibrosis increases, ultimately impairing liver function and advancing to end-stage liver disease (62, 63).

## 4 The integrative role of the IL-6 cytokine family in the MAFLD cytokine network

The progression of MAFLD, particularly the transition from NAFL to NASH and fibrosis, is intricately linked to a chronic inflammatory microenvironment (64). Within this pathological framework, members of the IL-6 cytokine family facilitate downstream signaling pathways via their common subunit gp130, thereby establishing a complex regulatory network among hepatocytes, immune cells such as macrophages and neutrophils, and HSCs. This network plays a central integrative role. Notably, lipid overload in hepatocytes constitutes a critical factor driving the transition from NAFL to NASH (3). Specific cytotoxic lipid species induce lipotoxicity and LIF facilitates the release of multiple cytokines by hepatocytes. IL-6, IL-11, OSM, and LIF enhance acute-phase liver responses, cause insulin resistance in liver cells, and drive macrophages to a pro-inflammatory M1 state, thus worsening liver inflammation (65, 66). Research has demonstrated that lipid toxicity-induced hepatocytes secrete exosomes enriched with microRNA-192-5p, which play a pivotal role in M1 macrophage activation and liver inflammation (67). Furthermore, IL-6, IL-11, and OSM activate HSCs and enhance the expression of fibrotic genes (68). Consequently, the IL-6 cytokine family, via its complex cytokine network originating from multiple cell types, facilitates the advancement of MAFLD and occupies a pivotal regulatory position in the cascade of”metabolic dysfunction-inflammatory response-fibrosis progression” progression.

## 5 IL-6 family’s role in MAFLD pathogenesis resource identification initiative

### 5.1 IL-6 family and lipid metabolism regulation

Lipid metabolism balance is crucial for energy supply, cell membrane integrity, and signaling. Its disruption is associated with metabolic disorders like obesity, atherosclerosis, and MAFLD (69). Fatty liver forms due to disrupted liver lipid metabolism and excessive triglyceride buildup in liver cells. This can be caused by increased fatty acid uptake and synthesis, decreased lipolysis, and reduced triglyceride or very low-density lipoprotein (VLDL) release (69). Hepatic lipids are regulated by complex interactions between external and internal factors, involving various cell types and multiple cytokines (70). The IL-6 family, including IL-6, IL-11, LIF, and OSM, plays a key role in MAFLD by disrupting lipid metabolism and causing abnormal lipid buildup.

When it comes to the regulation of lipid metabolism, acetyl-coenzyme A (CoA) carboxylase (ACC) plays a pivotal role by catalyzing the conversion of acetyl-CoA to malonyl-CoA. This reaction facilitates the incorporation of carbon into the *de novo* synthesis of fatty acids and serves as a key regulatory point in cellular lipid metabolism. ACC is essential for shifting from early lipid metabolism to glycolysis and remodeling in macrophages (71). Studies have demonstrated that upregulation of IL-6 expression leads to increased protein expression levels of sterol regulatory element-binding protein 1 (SREBP-1), fatty acid synthase (FASN), and ACC. This upregulation boosts protein synthesis and inhibits their expression in adipocytes, promoting fat production and resulting in increased lipid accumulation in non-adipose tissues such as the liver and muscle (72). Furthermore, IL-6 increases the serum expression levels of FAA, resulting in disruptions to the lipid metabolism system and inducing IR, thereby exacerbating liver injury (72, 73). Research shows that IL-6 from fat tissue influences liver insulin resistance by increasing Suppressor of Cytokine Signaling 3 (SOCS3) levels (73). SOCS3 overexpression in the liver leads to insulin resistance and increases SREBP-1 expression, which regulates fatty acid synthesis (74). These results suggest that IL-6 plays a role in fat production and insulin resistance in non-adipose tissues.

Hepatocytes exhibit elevated expression of IL11RA and secrete IL-11 in response to the accumulation of lipids. The autocrine activity of IL-11 is facilitated by reactive oxygen species (ROS) derived from NADPH Oxidase 4 (NOX4), as well as by the activation of Extracellular Signal-Regulated Kinase (ERK), c-Jun N-terminal Kinase (JNK), and cysteine-aspartate-specific kinase (CASK). This signaling cascade, involving ERK, JNK, and Cysteine Aspartate-Specific Protease 3 (caspase-3), results in compromised mitochondrial function and diminished fatty acid oxidation, ultimately impacting hepatocyte viability (75). Excess lipids in liver cells trigger IL-11 secretion, enhancing its autocrine action, which then upregulates NOX4 and boosts ROS production. This sequence of events impairs the oxidative capacity of hepatocyte mitochondria and disrupts fatty acid metabolism, culminating in the development of steatosis (75). New findings **suggest have** elucidated the mechanism by which IL-11 contributes to the progression from MAFLD to NASH. By developing a model of lipotoxicity-driven disease progression, researchers have demonstrated that lipid-accumulating hepatocytes secrete IL-11. This secretion induces metabolic dysfunction and hepatocyte cell death through an autocrine effect, while also activating HSCs and other hepatocytes via a paracrine mechanism (73). IL-11 triggers lipotoxic injury via hepatocyte-specific cis-signaling, influencing metabolic balance and offering insights into the progression from MAFLD to NASH (75).

Tumor tissues express high levels of LIF due to its complex role and specific action on cells and tissues. This molecule, secreted by tumors, promotes fat breakdown in cancerous areas, causing weight loss (76).LIF is crucial for regulating triglyceride (TG) metabolism in the liver, essential for maintaining lipid balance. LIF expression levels are significantly correlated with the pathological progression of MAFLD. At normal expression levels, LIF exerts a protective effect on hepatic lipid homeostasis by initiating the STAT3 signaling pathway, which inhibits the expression of SREBP-1c, thereby reducing TG accumulation in the liver (77). Conversely, LIF overexpression disrupts lipid homeostasis and diminishes hepatic *de novo* lipogenesis. Overexpression activates STAT3 signaling in hepatocytes, downregulating Peroxisome Proliferator-Activated Receptor Alpha (PPARα), reducing lipogenesis, and disrupting liver lipid balance (77, 78).

OSM serves multiple functions in hepatic lipid metabolism. Microglia and macrophages express it in response to prostaglandin E2 (PGE2) through a signaling pathway dependent on cAMP/PKA (79). In hepatocytes, OSM expression inhibits carnitine palmitoyltransferase-1 (CPT1), thereby preventing the entry of fatty acids into mitochondria for β-oxidation, which leads to their intracellular accumulation (80). Furthermore, OSM induces the expression of apolipoprotein B (ApoB), which, in conjunction with PGE2, inhibits the expression of microsomal transfer proteins. This inhibition results in impaired VLDL expression, further exacerbating triglyceride accumulation in the liver (81), a process intricately linked to the development of IR (80).

Furthermore, OSM facilitates the transition from fatty acid oxidation to Lipid buildup in hepatocytes, which is caused by the elevated expression of SOCS3 (82–84). Importantly, adipose tissue macrophages(ATMs) are pivotal in metabolic inflammation, serving as a primary source of OSMβ. M1 macrophages infiltrate adipose tissue via a chemokine-dependent mechanism, and their secretion of inflammatory mediators impairs insulin signaling (85). This impairment exacerbates hepatic gluconeogenesis when insulin signaling is entirely disrupted in the liver.

When key insulin signaling components that suppress liver gluconeogenesis are impaired, the remaining functional pathways promote liver lipogenesis (86). Insulin boosts the expression of SREBF-1, FAS, and SCD-1, increasing liver fatty acid production, which may lead to more fat accumulation in the liver (87). This adipose tissue inflammation within the liver establishes a self-perpetuating cycle with dysregulated hepatic lipid metabolism, culminating in the onset of metabolic dysregulation (85).

### 5.2 IL-6 family and insulin resistance

Insulin, a protein hormone produced by the β-cells of the pancreatic islets, primarily functions to lower blood glucose levels, promote the synthesis of glycogen, lipids, and proteins, and regulate glucose and lipid metabolism (88). When insulin action is impaired, the liver cannot effectively lower blood sugar, leading to insulin resistance. This results in reduced glycogen synthesis, increased gluconeogenesis, higher blood glucose levels, more lipid production, and ultimately, fat buildup in the liver (89).

Excess fatty acid production overwhelms fat tissue storage, causing endocrine dysfunction and abnormal fat buildup. This leads to lipotoxicity, which triggers liver inflammation and insulin resistance (90). Lipotoxicity facilitates the release of inflammatory mediators and exacerbates IR, while also intensifying lipolysis and further elevating FFA levels. The interaction between lipotoxicity, insulin resistance, and inflammation creates a harmful cycle that advances MAFLD to more severe stages. Saturated fatty acids(SFAs) activate IκB kinase-β(IKK-β), a key kinase in the NF-κB pathway, significantly increasing the production of the pro-inflammatory cytokine IL-6 (91). IL-6 subsequently activates the JNK signaling pathway and inhibits the insulin secretory function induced by IL-1. However, chronic elevation of IL-6 disrupts glucose homeostasis and fosters the advancement of IR (92). Besides the effects of irregular lipid metabolism, inflammation plays a significant role in contributing to IR. Specifically, IL-6 boosts the expression of SOCS3 by activating dual signaling pathways mediated by STAT3 and NF-κB (80). SOCS3 is considered an essential mediator in the negative regulation of insulin signaling pathways by inflammatory cytokines (93). It exerts its effects by attaching to insulin receptors and inhibiting the phosphorylation of insulin receptor substrate 1 (IRS-1) and IRS-2, thereby reducing insulin-dependent glucose transport (94). This inhibitory effect is particularly pronounced in the liver, leading to impaired hepatic insulin receptor signaling and decreased systemic insulin sensitivity.

The inhibition of adipocyte differentiation and the expansion of adipose tissue have been identified as significant pathogenic factors in IR (95). OSM, an inhibitor of adipogenesis, negatively impacts metabolic homeostasis by affecting metabolic processes through two pathways. It directly contributes to insulin resistance by inhibiting adipogenesis (96). Secondly, OSM plays a pivotal role in insulin signaling by modulating ATM polarization. Research indicates that in mice deficient in the OSM receptor β-subunit (OSMRβ-/-), ATMs are polarized towards the M1 phenotype, resulting in heightened inflammation within fat tissue (97). This polarization leads M1 macrophages to infiltrate adipose tissue through a chemokine-dependent process, releasing pro-inflammatory cytokines such as TNF-α and IL-1β, which inhibit insulin signaling and worsen IR (85).

In hepatic metabolic regulation, PGE2 from Kupffer cells may affect liver insulin resistance by disrupting SOCS3, which decreases insulin-driven glucose use (80). Secondly, PGE2 may modulate insulin resistance by influencing cytokine production in liver nonparenchymal cells (80). Specifically, PGE2 induces the secretion of the cytokine OSM by Kupffer cells, which in turn activates SOCS3 expression in hepatocytes via the STAT3 signaling pathway (80). Importantly, SOCS3 not only serves as a downstream target of the STAT3 signaling pathway but also further impairs insulin signaling by inhibiting Akt phosphorylation and glucokinase activity. This dual mechanism ultimately exacerbates insulin resistance (80).

### 5.3 IL-6 family’s role in hepatic inflammatory response

IL-6, IL-11, and OSM from the IL-6 cytokine family exhibit strong pro-inflammatory effects in the liver, particularly during the NASH stage. They enhance inflammatory signaling, leading to the release of pro-inflammatory mediators and activation of Kupffer cells, which exacerbates liver inflammation (6, 98, 99).

IL-6 plays a bifunctional role in hepatic pathology. Initial research indicated that IL-6 facilitates liver regeneration following partial hepatectomy (100). Conversely, IL-6 demonstrates pro-inflammatory characteristics during chronic liver injury (101). The onset and persistence of liver inflammation play key roles in the pathogenesis of NASH (102), a condition distinctly marked by hepatic inflammation and steatosis. Moreover, in patients, higher plasma IL-6 levels are associated with more severe NASH. The underlying mechanism involves several signaling pathways: (1) the classical IL-6/IL-6 receptor (IL-6R) interaction induces gp130 dimerization, subsequently activating the downstream STAT3 signaling pathway through JAK phosphorylation (103).

Specifically, IL-6 impedes IR signaling and modulates both the acute phase response and chronic inflammation by inhibiting the SOCS-3-SREBP-1C-fatty acyl coenzyme A (FA-CoA) pathway, which subsequently suppresses IR signaling (6). Concurrently, IL-6 promotes Kupffer cells activation and secretion of pro-inflammatory cytokines, including tumor necrosis TNF-α and IL-1β, thereby enhancing the inflammatory response in hepatocytes and deterioration of the NASH (104). Secondly, phosphodiesterase 4 (PDE4) aggravates the inflammatory response in NASH by degrading cyclic adenosine monophosphate (cAMP) and disrupting intracellular signaling, which further elevates IL-6 expression levels (105). Thirdly, the core of inflammatory signaling is characterized by the reversible phosphorylation of protein regulators and effector molecules, particularly MAPK (106). Among these, p38α plays a pivotal role, with macrophage p38α being instrumental in promoting hepatic steatosis and the inflammatory response by inducing the secretion of the pro-inflammatory cytokine IL-6 through the polarization of M1 macrophages.

IL-11 is predominantly synthesized by fibroblasts, and prior research has identified its involvement in processes such as pro-platelet production, suppression of inflammatory responses, and mucosal protection (107). In models of hepatic ischemia/reperfusion (I/R), IL-11 demonstrates anti-inflammatory properties and hepatoprotective effects (108). However, recent investigations have indicated that IL-11 adversely impacts hepatocyte function following severe liver injury, as evidenced by the promotion of hepatocyte steatosis and accelerated disease progression (109). Additional studies have shown that the stimulation of transforming growth TGFβ1 induces the production of IL-11 receptor alpha (IL-11RA) in hepatocytes, thereby activating the IL-11 signaling pathway (109). This signaling pathway not only exhibits cytotoxic effects but also enhances the infiltration of inflammatory cells and promotes the release of pro-inflammatory cytokines via the inflammatory signaling pathway, initiating an inflammatory cascade that ultimately drives disease progression (109).

### 5.4 The regulatory function of the IL-6 family in the progression of liver fibrosis

Liver fibrosis is a pathological condition marked by an imbalance in the production and breakdown of extracellular matrix components (110). This condition begins when injured hepatocytes release chemokines, attracting inflammatory cells and amplifying local inflammation (111). The coordinated activation of liver stellate cells, myofibroblasts, cholangiocytes, and macrophages contributes to the excessive buildup of harmful extracellular matrix components, driving liver fibrosis progression (112). Early-stage hepatic fibrosis can be reversed as part of the body’s repair process, but without timely treatment, it may progress to irreversible cirrhosis (113). This progression increases the risk of portal hypertension, hepatic failure, and HCC, leading to organ failure and death (114, 115).

Damage to hepatocytes leads to a rise in inflammatory cell infiltration and the release of pro-inflammatory cytokines, which are crucial in initiating liver fibrosis (111). IL-6, a key inflammatory mediator, activates the STAT3 pathway, worsening hepatocyte damage and significantly promoting HSC activation (116). HSCs, which are the primary effector cells in the inflammatory response and liver fibrosis, maintain a quiescent phenotype under normal physiological conditions but undergo a multidimensionally regulated activation process (117). Upon activation, HSCs transform into myofibroblast-like cells through multiple signaling pathways. Besides the usual STAT3 pathway, IL-6 aids this conversion by activating the MAPK and JAK/STAT pathways, particularly via p38 and ERK (118). Recent research shows that Fendrr, a long non-coding RNA, binds to STAT2 to enhance IL-6 expression, stimulating HSC via paracrine signaling and promoting liver fibrosis progression (117).

IL-11 plays a crucial role in MAFLD progression by promoting inflammation and fibrosis through a multicellular mechanism. It interacts with HSCs via the IL-11RA receptor in a paracrine or autocrine manner. Hepatocytes release IL-11 when stimulated by TGFβ1, which then activates HSCs paracrinally (75). Moreover, HSCs release significant IL-11 levels when exposed to pathological stimuli (109). IL-11’s pro-fibrotic effects are mainly facilitated by activation of the HSC/extracellular signal-regulated kinase (ERK) pathway, a process that facilitates the conversion of HSCs into myofibroblasts and promotes the secretion of collagen and other matrix proteins, thereby directly contributing to fibrosis (119).

**Figure 1:**
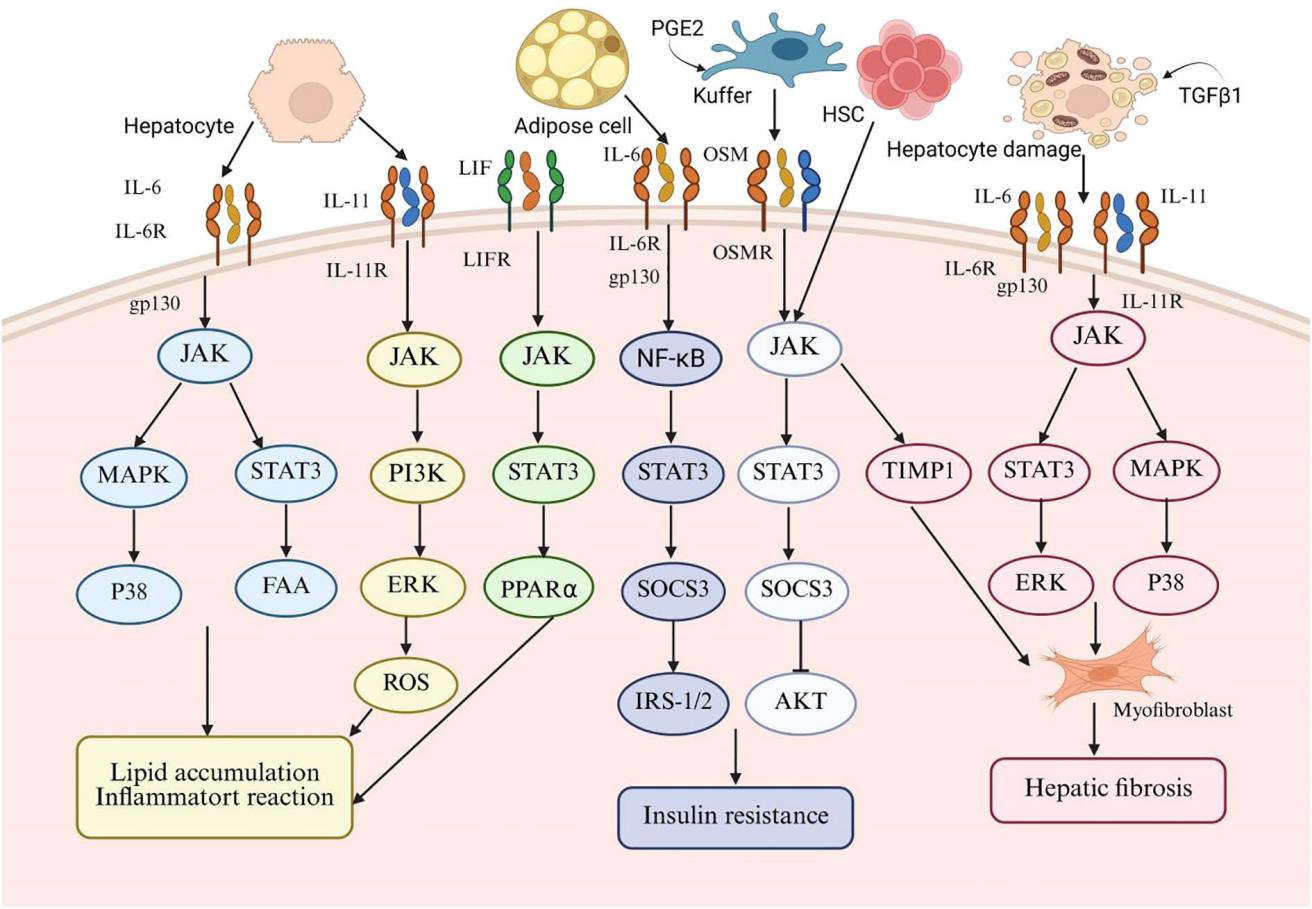
This schematic diagram systematically illustrates the central role of the interleukin-6 (IL-6) family of cytokines in the pathogenesis of metabolic-associated fatty liver disease (MAFLD). The diagram summarizes the key IL-6 family members (including IL-6, leukemia inhibitory factor (LIF), IL-11, and osteomodulin (OSM)) and the signaling pathways they mediate. For different target cells such as hepatocytes, adipocytes, Kupffer cells, and hematopoietic stem cells, the diagram highlights key molecular events including IL-6, the shared signal transduction receptor subunit gp130, the mitogen-activated protein kinase (MAPK) pathway, and the signal transduction and transcription activator 3 (STAT3) pathway. The sustained abnormal activation of these signaling pathways ultimately drives the formation of characteristic pathological processes of MAFLD, including hepatic lipid accumulation, chronic inflammatory response, insulin resistance, and hepatic fibrosis.

OSM, a cytokine released by Kupffer cells, shows varying expression in the liver. In a healthy liver, it aids development and regeneration, but in disease states, it promotes fibrosis (120). Research shows that OSM aids hepatocyte function recovery by inducing TIMP1 expression in acute liver injury but has pro-fibrotic effects in chronic liver disease (121). OSM enhances the articulation of TIMP1 and conversion of growth TGF-β through the activation of the JAK-STAT3 signaling pathway. TIMP1 subsequently inhibits fibrinolysis in HSCs and promotes fibrosis by inducing the expression of type I collagen (122). Furthermore, OSM has been observed to stimulate hepatic fibroblast migration and overexpression of pro-fibrotic factors in NAFLD (121). OSM facilitates fibrotic progression primarily through a synergistic mechanism involving the regulation of hepatic macrophages (HMs) and HSCs. This is evidenced by the upregulation of pro-fibrotic mediators like TGF-β and platelet-derived growth factor (PDGF) in HMs. Additionally, Fibrosis is indirectly influenced by OSM through the upregulation of TGF-β, PDGF, collagen, and TIMP1 in HSCs and myofibroblasts (MFs) (121). These findings indicate that OSM can induce hepatic fibrosis by modulating the interaction between HSCs and HMs and by promoting the migration of hepatic myofibroblasts (121) (**Figure 1**).

### 5.5 Other members of the IL-6 cytokine family and MAFLD

#### 5.5.1 IL-27

IL-27 is a multifunctional cytokine synthesized by antigen-presenting cells, playing a critical role in both inflammatory response and metabolic regulation. Its hepatoprotective mechanisms are primarily manifested in three domains: (1) Immunomodulation: The anti-inflammatory effects of IL-27 are achieved through its direct modulation of CD4^+^T cells and CD8^+^T cells, along with the enhancement of interleukin-10 and regulatory T cells (Tregs) activities (123). (2) Tissue Repair: IL-27 not only mitigates liver injury but also facilitates liver regeneration in instances of severe hepatic damage (68). (3) Metabolic Regulation: IL-27 reduces liver fat by decreasing endoplasmic reticulum stress, inhibiting fatty acid absorption, and boosting their oxidation through the AMPK/autophagy pathway (124). Recent research reveals a strong connection between MAFLD and hypothyroidism, highlighting that disrupted lipid metabolism in hypothyroidism leads to intrahepatic fat buildup, a key factor in MAFLD development (124, 125). This lipotoxicity notably exacerbates hepatic IR, thereby perpetuating a detrimental cycle of “lipid accumulation-IR.” Conversely, in this group, serum IL-27 levels were notably higher and showed a negative correlation with fasting blood glucose (FBG), the homeostasis model evaluation for insulin resistance, TG levels, and both subcutaneous and visceral fat content (126). The results indicate that IL-27 might have a protective role in slowing the progression of MAFLD. Consequently, in the future, the risk of MAFLD in patients with hypothyroidism could potentially be predicted by assessing serum IL-27 expression levels (126).

#### 5.5.2 CNTF

CNTF significantly influences fatty acid metabolism by promoting triglyceride release from the liver. It boosts insulin signaling molecule expression in 3T3-L1 adipocytes and reduces FAS and SREBP1 expression, both linked to MAFLD development (127). Research shows that CNTF can alleviate MAFLD, as recombinant CNTF improves obesity-related markers, lowers lipid levels, boosts insulin sensitivity, and significantly reduces liver injury and MAFLD in high-fat diet-induced obese rats (128).

#### 5.5.3 CLCF1

CLCF1 is a cholangiocyte-derived secreted factor that works with other cytokines to activate signaling pathways through the gp130/LIFR receptor complex, influencing various biological processes (129). Studies show that CLCF1 expression is significantly increased in the livers of diet-induced NASH mouse models and NASH patients. Additionally, high levels of CLCF1 in the liver help reduce inflammation and fibrosis by improving liver injury markers (34). The findings suggest that CLCF1 may protect against NASH development. LIFR, a key part of the CLCF1 receptor complex, is crucial for liver health during metabolic stress. In NASH, liver LIFR levels drop significantly, worsening the condition by disrupting CLCF1 signaling (34).

## 6 Prospects for IL-6 family clinical applications

MAFLD is a complex metabolic disorder driven by genetic, metabolic, and environmental factors. Its progression is mainly influenced by type 2 diabetes, insulin resistance, gut microbiota imbalance, genetics, diet, metabolism, and immune responses. A key mechanism in MAFLD’s advancement is the interaction between insulin resistance and inflammation, which together promote liver fat buildup and oxidative stress (2). Currently, there is no standardized treatment for MAFLD, so lifestyle changes and medication are the main options. Reducing high-sugar and high-fat foods can improve liver lipid metabolism, while regular exercise boosts fatty acid oxidation, promotes liver-protective autophagy, reduces liver fat, prevents liver cell death, and improves insulin sensitivity (130). Regarding pharmacological interventions, given the central role of IR, antidiabetic medications have emerged as a significant option in managing patients with clinically diagnosed Type 2 Diabetes Mellitus (T2DM) concomitant with MAFLD (2). Thiazolidinedione medications, including pioglitazone, have been demonstrated to effectively ameliorate insulin resistance in T2DM, as well as to mitigate hepatic steatosis and inflammation (131). Moreover, lipid-lowering agents, antihypertensive medications, and hepatoprotective compounds have demonstrated clinical efficacy and exhibit potential for further research in the management of MAFLD (2). Currently, drugs targeting hepatic *de novo* lipogenesis and glucose metabolism pathways are undergoing evaluation in Phase II or III clinical trials. These include inhibitors of stearoyl-CoA desaturase 1 (SCD1) and acetyl-CoA carboxylase (ACC), sodium-glucose cotransporter-2 (SGLT2) inhibitors, and fibroblast growth factor (FGF) analogues (132).

Presently, therapeutic approaches predominantly emphasize lifestyle modifications, with a limited array of pharmacological options available for MAFLD treatment. Consequently, it is imperative to identify and develop novel therapeutic targets and strategies (133). Recent studies suggest that Peroxisome Proliferator-Activated Receptors (PPARs) and their new ligands show promise as treatments for MAFLD. Currently in Phase II trials, these agents could greatly improve liver fibrosis reversal rates. PPARs, as nuclear receptors, are key in managing disrupted glucose and lipid metabolism and inflammation in MAFLD (132). Through a detailed study of disease mechanisms and targeting crucial pathways like oxidative stress with interventions such as angiotensin receptor blockers (ARBs) and antioxidants, new strategies are being developed to tackle and reverse steatosis at its source (132, 134–137).

This review highlights therapeutic strategies for MAFLD focusing on the IL-6 cytokine family, whose diverse roles in the disease can lead to varying therapeutic results depending on their activation or inhibition (68). The principal findings are as follows: 1) Targeting and inhibiting the IL-6 signaling pathway can mitigate liver damage and decelerate disease progression. Previous research has demonstrated that IL-6 gene knockout significantly reduces liver damage and inflammation in a methionine-choline-deficient (MCD) diet-induced NASH mouse model (138). Furthermore, elevated serum IL-6 levels are correlated with an increased risk of HCC (139)and have been identified as potential diagnostic markers for muscle wasting associated with cirrhosis and HCC (140). Targeting the IL-6/STAT3 pathway is a potential therapeutic strategy for HCC, involving interventions like IL-6 inhibitors (cetuximab, ADL518), IL-6R inhibitor (tocilizumab), JAK inhibitors (ruxolitinib), and direct STAT3 inhibitors (LLL12, C188-9) (140). Inhibition of the IL-6 downstream JAK/STAT3 signaling pathway has been shown to effectively suppress HCC cell proliferation and tumor growth (141, 142). In the non-neuronal cardiac cholinergic system (NNCCS), The vagus nerve reduces pro-inflammatory cytokines TNF and IL-6 via α7 nicotinic Ach receptors on Kupffer cells, crucial for liver glucose and energy metabolism regulation (143). 2) IL-11 and OSM are crucial for causing steatohepatitis and fibrosis. In NASH models, using anti-IL-11 antibodies or targeting IL-11RA can prevent or reverse these conditions (109). OSM reduces fatty acid content in hepatocytes by regulating key enzymes in hepatic lipid metabolism (85). Additionally, its receptor OSMRβ improves obesity-induced hepatic IR and steatosis by activating the JAK2/STAT3 signaling pathway (144). Macrophage-derived osteopontin (OPN, also known as SPP1) is notably enriched in the livers of MAFLD patients, exerting a protective effect by upregulating OSM. This upregulation enhances ARG2 expression through the STAT3 signaling pathway, leading to increased fatty acid oxidation (FAO) mediated by OPN, which effectively mitigates steatosis. This suggests that macrophage-derived SPP1 has the potential to prevent the progression of MAFLD (145). 3) CNTF and LIF exert protective effects in MAFLD. The administration of recombinant CNTF has been found to significantly ameliorate liver damage in patients with MAFLD (109). LIF is essential for regulating liver triglyceride balance, reducing fatty liver by binding to LIFR and activating the STAT3 pathway (77). Nonetheless, the overexpression of LIF in adipose tissue has been observed to reduce hepatic steatosis and insulin resistance, thereby slowing the progression of MAFLD (77). 4) Brown adipose tissue (BAT) is crucial for thermogenesis and energy metabolism. CLCF1 acts as a negative regulator of the PERK-ATF4 pathway, influencing the transcriptional activity of adenylate cyclase 3 (ADCY3) and consequently inhibiting thermogenesis. Knocking out CLCF1 in BAT boosts its activation and improves glucose and lipid metabolism, protecting mice from diet-induced obesity. This indicates that targeting CLCF1 signaling might be an effective treatment for obesity-related metabolic issues (146). Research indicates that the expression of CLCF1 is markedly upregulated following hepatic ischemia-reperfusion injury. MAFF (V-maf musculoaponeurotic fibrosarcoma oncogene homolog F) reduces hepatic cell apoptosis and inflammatory responses by activating the CLCF1 and STAT3 signaling pathways. This mechanism alleviates hepatic injury and presents a potential therapeutic target for liver protection (147). 5)Additionally, alterations in circulating IL-27 levels have been identified as a prospective therapeutic target for MAFLD (126). The pharmacological inhibition of the NOD-like receptor protein 3 (NLRP3) *in vivo* has been shown to reduce hepatic inflammation, hepatocyte damage, and subsequent fibrosis in MAFLD, suggesting a promising pharmacological treatment strategy (148).

## 7 Overview and future prospects

This paper offers an extensive overview of the existing research on the pathogenesis of MAFLD, with a particular emphasis on the pivotal role of the IL-6 cytokine family in the disease’s progression. Current studies suggest that the IL-6 family plays a crucial role in the development of MAFLD by interfering with lipid metabolism, promoting fat accumulation, inducing hepatocellular steatosis, facilitating fatty liver development, and expediting hepatic fibrosis, among other mechanisms. Despite the incomplete understanding of MAFLD pathogenesis, ongoing in-depth investigations into the mechanisms of action of IL-6 family members hold the potential to substantially inform and enhance current therapeutic strategies. A primary challenge facing contemporary research is the translational gap, where, despite the validation of several potential therapeutic targets in animal models, effective targeted therapies for humans remain elusive. This translational difficulty may arise from interspecies biological differences, the heterogeneity of target mechanisms, and the complexity associated with the various stages of disease progression. Research moving forward should emphasize the study of cytokines, including IL-31 and CTF1, whose mechanisms of action in MAFLD remain unclear. Emphasis should be placed on targeting specific cytokines and signaling pathways, as well as translating therapies that target gp130 signaling into clinical practice to further elucidate the synergistic effects arising from the crosstalk between these cytokines. These synergistic effects could be leveraged for multi-target therapies aimed at intervening in the progression of MAFLD. Beyond intrahepatic-specific therapeutic targets, extrahepatic targets, including the microbiome and the gut-hepatic signaling axis, suggest hopeful paths for the development of innovative treatment methods (68).

## Glossary

MAFLD: Metabolic-associated fatty liver disease
NASH: Non-alcoholic steatohepatitis
IL-6: Interleukin-6
NAFL: Non-Alcoholic Fatty Liver
IL-11: Interleukin-11
IL-27: Interleukin-27
IL-31: Interleukin-31
OSM: Oncostatin M
LIF: Leukemia inhibitory factor
CNTF: Ciliary neurotrophic factor
CTF1: Cardiotrophin 1
CLCF1: Cardiotrophin-like cytokine factor 1
gp130: Glycoprotein 130
JAK/STAT3: Janus kinase-signal transducer and activator of transcription 3
NF-κB: Nuclear factor kappa-light-chain-enhancer of activated B cells
PI3K/AKT: Phosphoinositide 3-kinase/Ak strain Transforming
MAPK: Mitogen-Activated Protein Kinase
p38 MAPK-PGC-1α: p38 Mitogen-activated protein kinase-Peroxisome proliferator-activated receptor γ coactivator-1α
AMPK/ULK1: Adenosinemonophosphate-activated Protein Kinase-Unc-51 Like Kinase 1
ERK-AMPK-mTORC1: Extracellular signal-Regulated kinase-AMP-Activated Protein Kinase-mechanistic Target of Rapamycin Complex 1
IL-1β: Interleukin-1 beta
UCP1: Uncoupling Protein 1
LIFR: Leukemia inhibitory factor receptor
OSMR: Oncostatin M receptor
MSC: Mesenchymal stem cell
MMPs: Matrix metalloproteinases
TIMP1: Tissue Inhibitor of Metalloproteinases 1
IL-31Rα: IL-31 receptor alpha
ALT: Alanine aminotransferase
AST: Aspartate aminotransferase
CT: Computed tomography
MRI: Magnetic resonance imaging
FFAs: Hepatic free fatty acids
IR: Insulin resistance
HSCs: Hepatic stellate cells
VLDL: Very low-density lipoprotein
ACC: Acetyl-coenzyme A carboxylase
SREBP-1: Sterol regulatory element-binding protein 1
FASN: Fatty acid synthase
SOCS3: Suppressor of Cytokine Signaling 3
ROS: Reactive oxygen species
NOX4: NADPH Oxidase 4
ERK: Extracellular Signal-Regulated Kinase
CASK: Cysteine-aspartate-specific kinase
Caspase-3: Cysteine Aspartate-Specific Protease 3
TG: Triglyceride
PPARα: Receptor Alpha
PGE2: Prostaglandin E2
CPT1: Carnitine palmitoyltransferase-1
ApoB: Apolipoprotein B
ATMs: Adipose tissue macrophages
SFAs: Saturated fatty acids
FBG: Fasting blood glucose
IKK-β: κB kinase-β
IRS-1: Insulin receptor substrate 1
OSMRβ-/-: OSM receptor β-subunit
FA-CoA: Fatty acyl coenzyme A
PDE4: Phosphodiesterase 4
cAMP: Cyclic adenosine monophosphate
I/R: Ischemia/reperfusion
IL-11RA: IL-11 receptor alpha
ERK: Extracellular signal-regulated kinase
HMs: Hepatic macrophages
PDGF: Platelet-derived growth factor
MFs: Myofibroblasts
FBG: Fasting blood glucose
T2DM: Type 2 Diabetes Mellitus
ARBs: Angiotensin receptor blockers
